# HIV-1 Tat inhibits EAAT-2 through AEG-1 upregulation in models of HIV-associated neurocognitive disorder

**DOI:** 10.18632/oncotarget.16485

**Published:** 2017-03-22

**Authors:** Xiang Ye, Yu Zhang, Qiping Xu, Honghua Zheng, Xiaoyan Wu, Jinhua Qiu, Zhou Zhang, Wei Wang, Yiming Shao, Hui Qin Xing

**Affiliations:** ^1^ Department of Pathology, Fujian Provincial Key Laboratory of Neurodegenerative Disease and Aging Research, Institute of Neuroscience, Basic Medicine, Medical College, Xiamen University, Xiamen, Fujian, China; ^2^ State Key Laboratory for Infectious Disease Prevention and Control, Collaborative Innovation Center for Diagnosis and Treatment of Infectious Diseases, National Center for AIDS/STD Control and Prevention, Chinese Center for Disease Control and Prevention, Beijing, China; ^3^ Institute of Laboratory Animal Sciences of Chinese Academy of Medical Science, Beijing, China

**Keywords:** AEG-1, EAAT-2, HIV-1 Tat, HIV-associated neurocognitive disorder, PI3-K

## Abstract

During HIV-associated neurocognitive disorder (HAND), decreasing in excitatory amino acid transporter 2 (EAAT-2) in astrocyte plasma membranes leads to elevated levels of extracellular glutamate and, in turn, neuronal apoptosis. We used immunohistochemistry, western blot, qRT-PCR, and RNA interference to elucidate the molecular mechanisms underlying the decreased EAAT-2 expression during HAND at the tissue and cellular levels. We used simian immunodeficiency virus-human immunodeficiency virus chimeric virus (SHIV)-infected macaques as an *in vivo* model of HAND. Our results show that EAAT-2 expression was decreased in the cerebral cortex, while AEG-1 expression was increased, and the expression levels of these proteins were negatively correlated. I*n vitro* analyses showed that HIV-1 Tat inhibited EAAT-2 expression by inducing overexpression of AEG-1. More specifically, HIV-1 Tat increased AEG-1 expression *via* the PI3-K signaling pathway, while increasing EAAT-2 inhibition by YinYan-1 (YY-1) *via* the NF-κB signaling pathway. These results warrant testing AEG-1 as a potential therapeutic target for treating HAND.

## INTRODUCTION

HIV-associated neurocognitive disorder (HAND) is a common syndrome characterized by cognitive impairment, decreased exercise capacity, and behavioral changes. According to statistics from the World Health Organization, 1/3-2/3 of Acquired Immune Deficiency Syndrome (AIDS) patients suffer from HAND, with an increasing morbidity of HAND in recent years [1, 2]. HAND severely impacts patient quality of life and can be life threatening. The molecular mechanisms underlying HAND-derived impairments remain poorly understood.

Recent studies have shown that HAND results from direct toxic damage of nerve cells by HIV-1, gpl20, gp41, and transactivator of transcription (tat), as well as from immune reactions and inflammatory injury to brain tissue caused by cytokines, such as tumor necrosis factor-α (TNF-α), interleukin-lβ (IL-lβ), and interieukin-6 (IL-6), which are released by viral protein-activated microglia/macrophage [3, 4]. These toxic cytokines stimulate microglia to secrete large amounts of excitatory neurotransmitter glutamate (Glu), which results in a continuous increase of extracellular Glu concentration. Previous results from our group showed that low expression of excitatory amino acid transporter 2 (EAAT-2) on the astrocytic membrane may promote high levels of Glu in the extracellular synaptic cleft and subsequent neuronal death [5, 6]. A continuously increasing concentration of extracellular Glu leads to activation of the n-methyl-d-aspartate receptor (NMDAR), followed by neuronal excitatory toxic injury [7]. However, the molecular mechanisms responsible for downregulating EAAT-2 expression are not yet clear.

According to previous reports, HIVgp120 (as well as TNF-α) upregulates expression of astrocyte-elevated gene-1 (AEG-1) on the cell surface of primary human fetal astrocytes (PHFA) cultured *in vitro* [8], further decreasing EAAT-2 expression. However, the correlation between AEG-1 and EAAT-2 expression patterns and the specific molecular mechanisms involved in HAND require further analysis.

The AEG-1 gene is located on chromosome 8q22, and encodes for a protein with a molecular weight of 64 kDa. It is expressed in the periphery of the cell nucleus and the endoplasmic reticulum [9]. Recent studies have shown AEG-1's role as an oncogene in multiple malignant tumors [10]. Nevertheless, very little is known about the molecular mechanisms implicating AEG-1 in HAND. According to tumor cell studies, AEG-1 positively activates NF-κB to downregulate EAAT-2 expression [11, 12], phosphorylates serine/threonine kinase (Akt), and contributes to infiltration and metastasis of tumor cells [13, 14, 15]. Interestingly, activation of the phosphatidylinositol-3-kinase/threonine kinase (PI3K/Akt) signaling pathway can upregulate AEG-1 expression in astrocytes [16]. The present study aimed to further analyze the correlation between AEG-1 and EAAT-2 expression patterns and to uncover specific molecular mechanisms underlying HAND at the tissue and cellular level. To this end, we used SHIV-infected macaques animal models and performed cell biology experiments, as well as double-labeling immunofluorescence, western blot, qRT-PCR, and RNA interference.

## RESULTS

### Viral RNA loads in SHIV-infected macaques

Viral RNA loads in peripheral blood at the time of autopsy from 8 SHIV-infected macaques are summarized in Table 1. A total of eight macaques (*#E1-E8*) were infected with SHIV-_SF162..P4_. All SHIV-infected macaques showed high viral loads, especially #E1 and #E8, as well as weight loss prior to death and autopsy. In a previous study, we demonstrated that there are differences in viral RNA load among rhesus macaques infected with the same virus (SIVmac239 or SHIV-RT). However, there was no correlation between viral RNA load in plasma and EAAT-2 expression in the cerebral cortex [6]. In this study, there was also no correlation between viral RNA load in plasma and EAAT-2 and AEG-1 expression in the cerebral cortex of SHIV_-SF162.P4_ infected rhesus macaques.

**Table 1:** Clinical data of the macaques examine in this study

Animal No.	Sex	Age at virus inoculation (weeks)	Age at Death (weeks)	Duration of infection (weeks)	Viral inoculums	Viral RNA load in plasma at autopsy (copies/ml)	Clinical information
E1 E2 E3 E4 E5 E6 E7 E8	F M F M F M F M	40 40 40 40 40 40 40 40	68 68 68 68 68 68 68 68	28 28 28 28 28 28 28 28	SHIV-_SF162.P4_ SHIV- _SF162.P4_ SHIV- _SF162.P4_ SHIV- _SF162.P4_ SHIV- _SF162.P4_ SHIV- _SF162.P4_ SHIV- _SF162.P4_ SHIV- _SF162.P4_	7930000 1000000 1660000 2290000 1340000 2050000 4970000 9650000	Body weight loss and morbid Body weight loss and morbid Body weight loss and morbid Body weight loss and morbid Body weight loss and morbid Body weight loss and morbid Body weight loss and morbid Body weight loss and morbid
10 11 12 13	F M M M		156 210 209 253		Control Control Control Control		

### Association between decreased EAAT-2 and neuronal apoptosis in the cerebral cortex of SHIV-infected macaques

**Figure 1:**
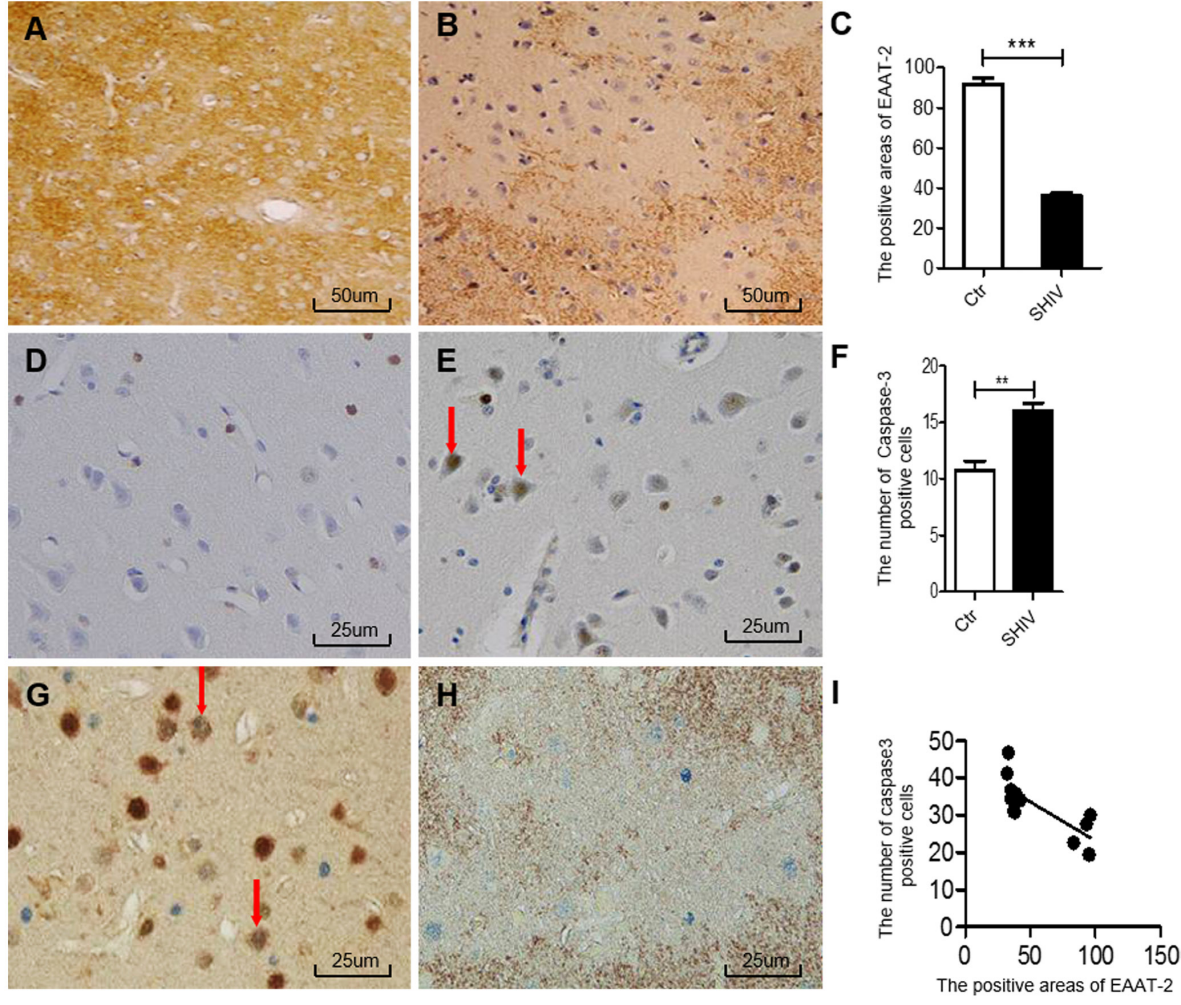
Association between decreased EAAT-2 and neuronal apoptosis in the cerebral cortex of SHIV-infected macaques **A**.-**B**. Decreased EAAT-2 expression in the cerebral cortex of SHIV-infected macaques (right) and controls (left). **C**. Statistical analysis of A and B (***P < 0.001). **D**.-**E**. The number of cleaved-caspase-3-positive cells in the cerebral cortex of SHIV-infected macaques (right) is increased compared with controls (left). **F**. Statistical analysis of D and E (**P < 0.01). **G**. Neuronal apoptosis in the cerebral cortex of SHIV-infected macaques as shown by NeuN (brown) and cleaved-caspase-3 (blue) double immunohistochemical staining. **H**. Neuronal apoptosis in areas with decreased EAAT-2 expression according to cleaved-caspase-3 (blue) and EAAT-2 (brown) double immunohistochemical staining. **I**. Statistical analysis of H demonstrates that areas with EAAT-2 expression correlate negatively with the number of cleaved-caspase 3-positive cells (*P < 0.05 and R^2^ = 0.5861). Ctr: uninfected group; SHIV: SHIV-infected group.

We used immunohistochemical staining to analyze the relationship between decreased EAAT-2 levels and neuronal apoptosis. Expression of EAAT-2 in the frontal cortex of SHIV-infected macaques (Figure 1B) was decreased compared with controls (Figure 1A), and the difference was statistically significant (Figure 1C). Additionally, the number of cleaved-caspase-3-positive cells in the frontal cortex of SHIV-infected macaques (Figure 1E) was increased compared with the controls (Figure 1D), with a statistically significant difference (Figure 1F). Double-labeled immunohistochemistry for cleaved-caspase-3 and NeuN revealed double labeling in the frontal cortex of SHIV-infected macaques, which is indicative of neuronal apoptosis (Figure 1G). The number of cleaved-caspase-3-positive cells was also increased in the area of decreased EAAT-2 expression (Figure 1H), with a statistically significant negative correlation (Figure 1I, *p < 0.05, R2 = 0.5861). These results demonstrated that decreased EAAT-2 expression correlates with neuronal apoptosis in the frontal cortex of SHIV-infected macaques.

### Increased AEG-1 and decreased EAAT-2 expression in the cerebral cortex of SHIV-infected macaques

**Figure 2:**
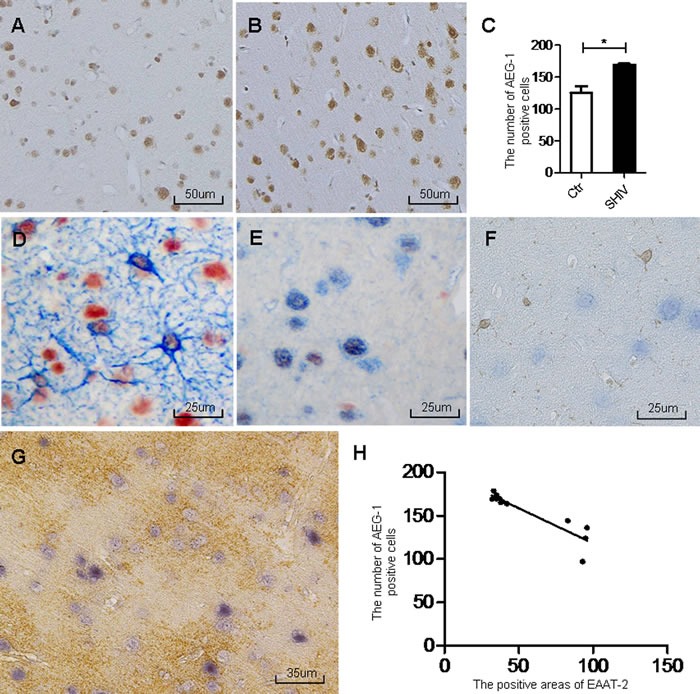
Increased AEG-1 expression in area of decreased EAAT-2 in the cerebral cortex of SHIV-infected macaques **A**.-**B**. AEG-1 expression is increased in the cerebral cortex of SHIV-infected macaques (right) compared with controls (left). **C**. Statistical analysis of A and B (*P < 0.05). **D**. Double immunohistochemical staining shows AEG-1 expression primarily in astrocytes of the cerebral cortex of SHIV-infected macaques (AEG-1 is red, GFAP is blue). **E**. Partial expression in neurons (AEG-1 is red, NeuN is blue). **F**. very little expression in microglia (AEG-1 is blue, Iba-1 is brown). **G**. Increased number of AEG-1-positive cells in the area of decreased EAAT-2 expression according to AEG-1 (blue) and EAAT-2 (brown) double immunohistochemical staining. **H**. statistical analysis of G demonstrates that the area of EAAT-2 expression negatively correlates with the number of AEG-1-positive cells (*P < 0.05, R^2^ = 0.8327). Ctr: uninfected group; SHIV: SHIV-infected group.

Immunohistochemistry showed increased AEG-1 expression in the frontal cortex of SHIV-infected macaques (Figure 2B) compared with the controls (Figure 2A), with a statistically significant difference (Figure 2C). We then performed double-labeling immunohistochemistry experiments for AEG-1 in combination with GFAP, NeuN, or Iba-1. The results showed that AEG-1 was mainly expressed in astrocytes of the cerebral cortex in SHIV-infected macaques (Figure 2D), and only partially expressed in neurons (Figure 2E), and barely expressed in microglia (Figure 2F). Finally, we performed double-labeling immunohistochemistry for AEG-1 and EAAT-2, showing that the number of AEG-1-positive cells was increased in the area of decreased EAAT-2 expression (Figure 2G), and statistical analysis of the relationship between the number of AEG-1-positive cells and positive EAAT-2 expression area revealed a significant negative correlation (Figure 2H).

### HIV-1 Tat decreases EAAT-2 expression and increases AEG-1 expression *in vitro*

**Figure 3:**
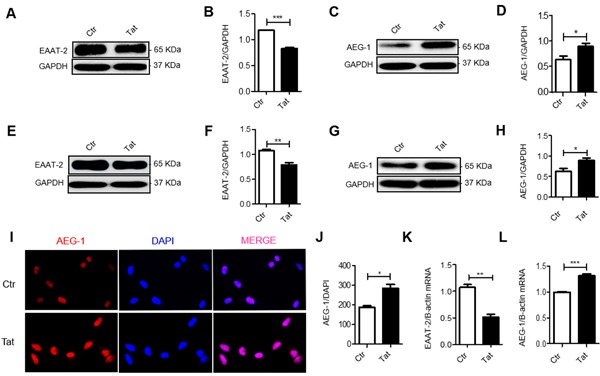
HIV-1 Tat decreases EAAT-2 expression and increases AEG-1 expression *in vitro* Primary mouse astrocytes and U87 cells were treated with or without recombined HIV-1 Tat (100 ng/ml) for 48 h. **A**.-**B**. EAAT-2 expression was decreased in HIV-1 Tat treated primary cultured mouse astrocytes. **C**.-**D**. AEG-1 expression was increased in HIV-1 Tat treated primary cultured mouse astrocytes. **E**.-**F**. EAAT-2 expression was decreased in HIV-1 Tat treated U87 cells. **G**.-**H**. AEG-1 protein expression was increased in HIV-1 Tat treated U87 cells. **I**.-**J**. AEG-1 expression was detected by fluorescence was increased in HIV-1 Tat treated U87 cells (original magnification ×400). **K**. EAAT-2 mRNA expression was decreased in HIV-1 Tat treated U87 cells. **L**. AEG-1 mRNA expression was increased in HIV-1Tat treated U87 cells. Data are plotted as mean ± SEM (*n* = 3). *P < 0.05; **P < 0.01; ***P < 0.001. Ctr: untreated group; Tat: HIV-1 Tat treatment group.

We next analyzed the relationship between AEG-1 and EAAT-2 expression at the cellular level. Primary cultured mouse astrocytes and the U87 glioma cell line were treated with recombinant HIV-1 Tat for 48 h. A separate control group was not treated. Results showed increased AEG-1 protein, mRNA and fluorescence expression (Figure 3C, 3D, 3G, 3H, 3I, 3J and 3L) and decreased EAAT-2 protein and mRNA expression (Figure 3A, 3B, 3E, 3F, and 3K) in the HIV-1 Tat treated group. These results demonstrated that HIV-1 Tat increased AEG-1 and decreased EAAT-2 at the cellular level.

### HIV-1 Tat regulates EAAT-2 through AEG-1 in the U87 glioma cell line

**Figure 4:**
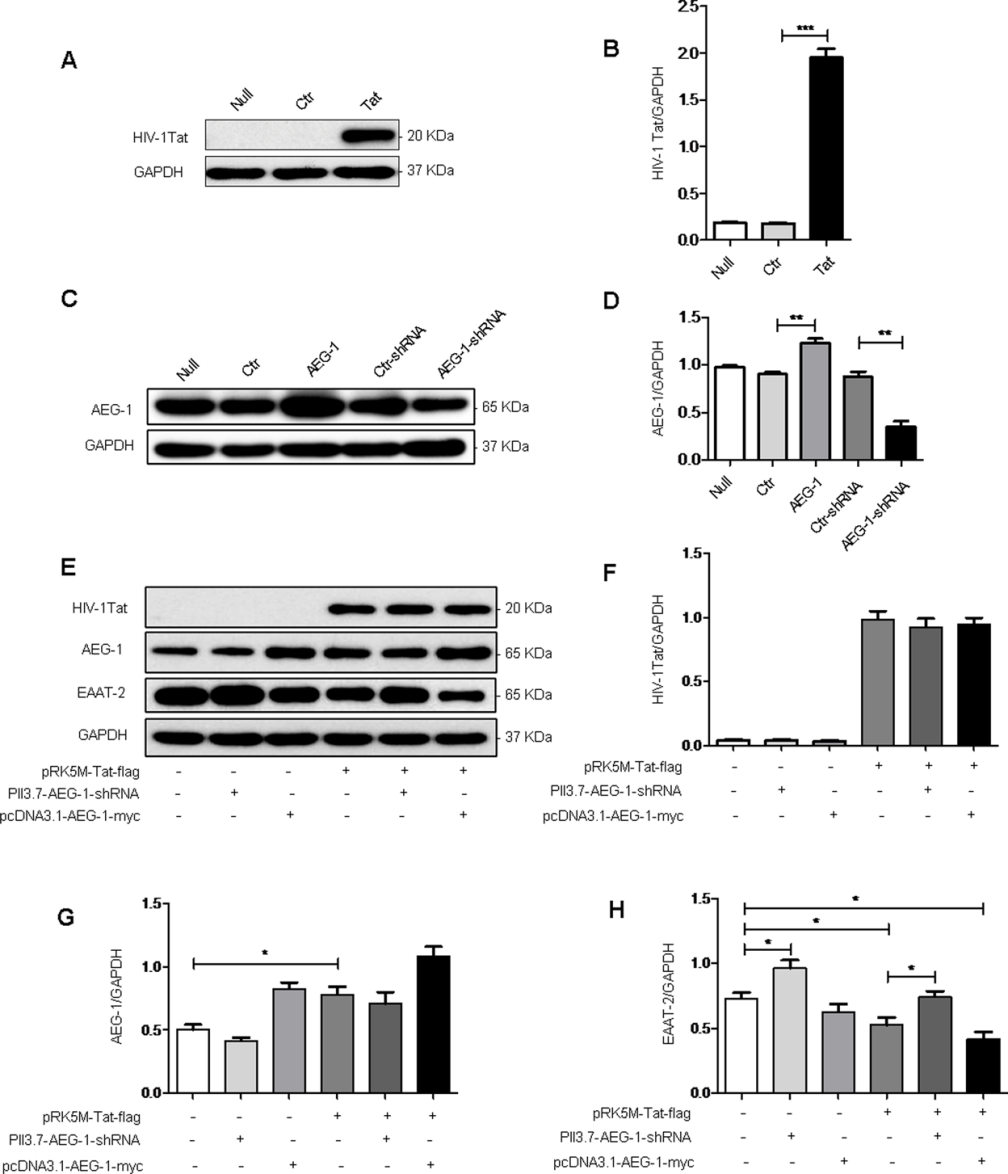
HIV-1 Tat downregulates EAAT-2 in U87 cells *via* AEG-1 in U87 cells U87 cells were transfected with pRK5M-flag (3 μg), pRK5M-Tat-flag (3 μg), pcDNA3.1-myc (3 μg), pcDNA3.1-AEG-1-myc (3 μg), Pll3.7-AEG-1-scamble (3 μg), or Pll3.7-AEG-1-shRNA (3 μg) for 48 h. **A**.-**B**. HIV-1 Tat protein expression was increased in pRK5M-Tat-flag-transfected cells compared with pRK5M-flag-transfected cells. **C**.-**D**. AEG-1 protein expression was increased in pcDNA3.1-AEG-1-myc-transfected cells compared with pcDNA3.1-myc-transfected cells. Also, AEG-1 protein expression was decreased in Pll3.7-AEG-1-shRNA-transfected cells compared with Pll3.7-AEG-1-scamble-transfected cells. **E**.-**H**. EAAT-2 protein expression was decreased after overexpression of HIV-1 Tat or AEG-1 in U87 cells, suggesting that there is a synergistic effect on EAAT-2 regulation. The ability of HIV-1 Tat to downregulate EAAT-2 was inhibited by AEG-1 knockdown in conjunction with HIV-1 Tat overexpression. Data are plotted as mean ± SEM (*n* = 3). *P < 0.05; **P < 0.01; ***P < 0.001. Null: untreated; Ctr: pRK5M-flag or pcDNA3.1-myc; Tat: pRK5M-Tat-flag; AEG-1: pcDNA3.1-AEG-1-myc; Ctr-shRNA: Pll3.7-AEG-1-scamble; AEG-1-shRNA: Pll3.7-AEG-1-shRNA.

Three distinct plasmids were constructed *in vitro*, namely pRK5M-Tat-flag, pcDNA3.1-AEG-1-myc, and Pll3.7-AEG-1-shRNA. To further investigate the relationships between HIV-1 Tat, AEG-1, and EAAT-2, we transfected U87 cells with each of these plasmids separately for 48 h and protein expression was measured by western blot. Results showed increased HIV-1 Tat expression in U87 cells following transfection with pRK5M-Tat-flag (Figure 4A and 4B). AEG-1 expression was also increased following transfection with pcDNA3.1-AEG-1-myc (Figure 4C and 4D). Conversely, AEG-1 expression decreased in U87 cells following transfection with Pll3.7-AEG-1-shRNA (Figure 4C and 4D).

Next, we transfected U87 cells with the three aforementioned plasmids, simultaneously. EAAT-2 protein expression significantly decreased following overexpression of AEG-1 in combination with HIV-1 Tat, AEG-1, or HIV-1 Tat, suggesting that there's a synergistic effect on EAAT-2 regulation (Figure 4E, 4F, 4G and 4H). However, the ability of HIV-1Tat to downregulate EAAT-2 was inhibited by AEG-1 knockdown while simultaneously overexpressing HIV-1 Tat (Figure 4E, 4F, 4G and 4H). These results demonstrated that HIV-1 Tat downregulated EAAT-2 expression through AEG-1 in U87 cells.

### HIV-1 Tat upregulates AEG-1 *via* the PI3-K signaling pathway

**Figure 5:**
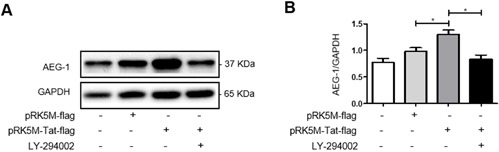
HIV-1 Tat upregulates AEG-1 in U87 cells *via* the PI3-K signaling pathway U87 cells were transfected with pRK5M-Tat-flag (3 μg) only or pRK5M-flag (3 μg) only for 48 h, or were treated with the PI3-K signaling pathway inhibitor LY-294002 (6.14 μg/ml) for 24 h following transfection with pRK5M-Tat-flag (3 μg) for 24 h. **A**.-**B**. AEG-1 protein expression was increased following transfection with pRK5M-Tat-flag only for 48 h. The ability of HIV-1 Tat to upregulate AEG-1 was inhibited by addition of LY-294002 following transfection with pRK5M-Tat-flag. Data are plotted as mean ± SEM (*n* = 3). *P < 0.05.

Next, we investigated the signaling pathway underlying the upregulation of AEG-1 induced by HIV-1 Tat. U87 cells were treated with the PI3-K signaling pathway inhibitor LY-294002 for 24 h following transfection with pRK5M-Tat-flag for 24 h. Total cellular protein was extracted to measure AEG-1 expression by western blot. The results showed increased AEG-1 protein expression following transfection with pRK5M-Tat-flag for 48 h (Figure 5A and 5B). However, the ability of HIV-1 Tat to upregulate AEG-1 was inhibited by the addition of LY-294002 following transfection with pRK5M-Tat-flag (Figure 5A and 5B). These results demonstrated that HIV-1 Tat increased AEG-1 *via* the PI3-K signaling pathway in U87 cells.

### AEG-1 regulates YY-1 expression *via* the NF-κB signaling pathway and inhibits EAAT-2 expression

**Figure 6:**
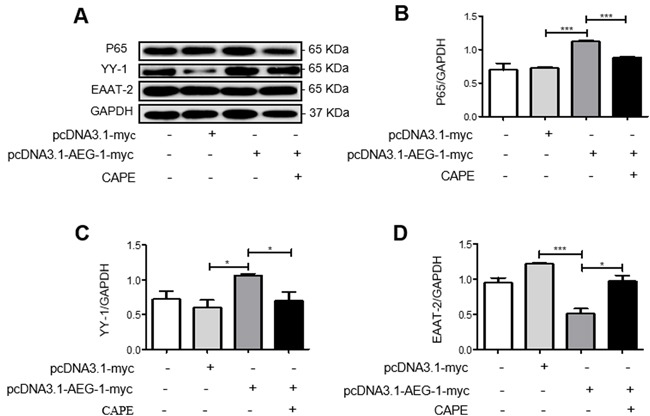
AEG-1 upregulates YY-1 expression and inhibits EAAT-2 expression in U87 cells *via* the NF-κB signaling pathway U87 cells were transfected with pcDNA3.1-AEG-1-myc (3 μg) only for 48 h or with the NF-κB signaling pathway inhibitor CAPE (30 μg/ml) for 24 h following transfection with pcDNA3.1-AEG-1-myc (3 μg) for 24 h. **A**.-**D**. P65 and YY-1 protein expressions were increased while EAAT-2 protein expression decreased following transfection with pcDNA3.1-AEG-1-myc. The ability of AEG-1 to upregulate P65 and YY-1, and to downregulate EAAT-2, was inhibited by the addition of CAPE following transfection with pcDNA3.1-AEG-1-myc. Data are plotted as mean ± SEM (*n* = 3). *P < 0.05; **P < 0.01; ***P < 0.001.

We also investigated the signaling pathways underlying the downregulation of EAAT-2 by AEG-1 using western blot. The expression of P65 and YY-1 proteins increased while EAAT-2 protein expression decreased following transfection of U87 cells with pcDNA3.1-AEG-1-myc only (Figure 6A, 6B, 6C and 6D). However, the ability of AEG-1 to upregulate YY-1 and P65 expression was inhibited by the addition of the NF-κB signaling pathway inhibitor CAPE following transfection with pRK5M-Tat-flag (Figure 6A, 6B and 6C). Protein expression of EAAT-2 also increased with decreased YY-1 expression (Figure 6A, 6C and 6D). These results demonstrated that AEG-1 upregulated YY-1 expression and inhibited EAAT-2 expression *via* the NF-κB signaling pathway.

## DISCUSSION

Neuronal apoptosis is pathological hallmark of HAND. Immune reactions, cytokine-induced neurotoxicity, and abnormal astrocyte function are also characteristic of HAND [3, 17]. In previous studies, we demonstrated that reduced EAAT-2 expression correlated with neuronal apoptosis in autopsies of HAND patients and SIV-infected macaques [5, 6]. A previous report showed a large amount of glutamate aggregation in the neuropil of the frontal cortex in autopsies of HAND patients, as well as high concentrations of glutamate in the cerebrospinal fluid [18]. Excessive concentrations of glutamate in the neuropil result in neurotoxicity and subsequent neuronal damage. Under normal circumstances in the brain, glutamate is released from the presynaptic membrane and it then passes through the synaptic cleft and is absorbed by the postsynaptic membrane. This process is largely dependent on EAAT-2 in the astrocytic membrane. If the expression or function of EAAT-2 is blocked and the enzymes do not exist to create glutamate *in vivo*, there will be excessive accumulation in the neuropil, thereby leading to neurotoxicity and ultimately neuronal death. However, the molecular mechanisms underlying decreased EAAT-2 expression remain unclear. Distinct pathological changes have been reported to occur in the frontal cerebral cortex of HAND autopsy specimens [19]. Therefore, we selected the frontal cortex region from SHIV-infected macaques for immunohistochemical staining. Our results showed reduced EAAT-2 expression in the cerebral cortex of SHIV-infected macaques, which positively correlated with neuronal apoptosis. Other researcher also demonstrated that reduced EAAT-2 expression would result in large amounts of glutamate aggregating in the synaptic cleft [20] and inducing neuronal apoptosis [21]. Accordingly, we hypothesized that reduced EAAT-2 expression leads to excitatory neurotoxicity and neuronal apoptosis.

AEG-1 expression has been shown to negatively correlate with EAAT-2 expression in the brains of glioma patients. Excessive AEG-1 levels in cultured glioma cells lead to reduced EAAT-2 expression and modify glutamate uptake, leading to neuronal apoptosis and neurodegenerative disease [22]. Here, results from our experiments showed increased AEG-1 expression in area with reduced EAAT-2 expression in the cerebral cortex of SHIV-infected macaques, suggesting that reduced EAAT-2 expression correlated with increased AEG-1 expression in HAND. However, the underlying mechanisms for such increased AEG-1 expression remain unknown. A previous study reported that AEG-1 expression is increased in Tat transgenic mice [23], suggesting that there is a relationship between Tat and AEG-1. Therefore, our experiments focused on testing for regulatory relationships between HIV-1 Tat, AEG-1, and EAAT-2.

HIV-1 Tat is a protein secreted by HIV and is commonly present in cerebrospinal fluid and serum of HIV-infected patients. HIV-1 Tat has been shown to promote the development of human immunodeficiency virus-related encephalitis (HIVE) [24–28]. HIV-1 Tat also stimulates neurons to release glutamate and activates microglia and induces release of glutamate *in vitro* and *in vivo* [29]. Therefore, we hypothesized that HIV-1 Tat also increased glutamate concentrations in the neuropil by inhibiting EAAT-2 expression during HAND pathogenesis. To test this, we treated U87 cells with recombined HIV-1 Tat and found that EAAT-2 mRNA and protein levels were reduced while AEG-1 mRNA and protein levels were increased. Results from our *in vivo* experiments showed increased AEG-1 expression in cerebral cortical area with reduced EAAT-2 expression in SHIV-infected macaques, suggesting that HIV-1 Tat inhibited EAAT-2 expression by upregulating AEG-1. Therefore, we transfected U87 cells with pRK5M-Tat-flag, pcDNA3.1-AEG-1-myc, and Pll3.7-AEG-1-shRNA. Our results showed that overexpression of HIV-1 Tat or AEG-1 resulted in reduced EAAT-2 protein expression, and that HIV-1 Tat and AEG-1 exhibited synergistic effects on EAAT-2 inhibition. However, when HIV-1 Tat was overexpressed in conjunction with AEG-1 interference, the ability of HIV-1 Tat to downregulate EAAT-2 was inhibited. These results suggested that HIV-1 Tat downregulated EAAT-2 expression by upregulating AEG-1. Therefore, we conducted further analyses on the relevant signaling pathways involved in this process.

AEG-1 is upregulated in NSC34 cells following activation of the PI3-K signaling pathway [16]. Therefore, we hypothesized that HIV-1 Tat upregulated AEG-1 expression in astrocytes *via* the PI3-K signaling pathway. Indeed, transfection of U87 cells with pRK5M-Tat-flag resulted in increased AEG-1 expression. On the other hand, if the cells were treated with the PI3-K inhibitor LY-294002 following transfection with pRK5M-Tat-flag, the ability of HIV-1 Tat to upregulate AEG-1 was suppressed. These results suggested that HIV-1 Tat upregulated AEG-1 expression *via* the PI3K signaling pathway.

Previous studies have shown that TNF-α induces AEG-1 expression in astrocytes, and that increased AEG-1 exerts its biological functions by binding with the NF-κB subunit P65. Additionally, as a type of transcriptional repressor protein in the NF-κB downstream pathway, YY-1 inhibits EAAT-2 expression [30]. Therefore, we hypothesized that the classical NF-κB/YY-1 pathway participates in AEG-1 regulation of EAAT-2. Therefore, we transfected U87 cells with pcDNA3.1-AEG-1-myc. Our results showed increased YY-1 and P65 expression and reduced EAAT-2 expression. However, when we treated U87 cells with the NF-κB inhibitor CAPE followed by transfection with pcDNA3.1-AEG-1-myc, the ability of AEG-1 to upregulate YY-1 and P65 was inhibited while EAAT-2 expression increased. These results suggested that AEG-1 upregulated YY-1 expression *via* the NF-κB signaling pathway, subsequently downregulating EAAT-2.

Results from our study show for the first time that HIV-1 Tat upregulated AEG-1 expression *via* the PI3-k signaling pathway, and that upregulated AEG-1 induced YY-1 expression *via* the NF-κB signaling pathway, thereby inhibiting EAAT-2 expression during HAND. These results could contribute to the development of treatment strategies and the discovery of therapeutic targets for HAND.

## MATERIALS AND METHODS

### SHIV-infected animals

SHIV-_SF162.P4_ is a T-lymphocyte-tropic virus, the pathogenic properties of which have been previously described [31, 32]. This virus includes the env, rev, and vpu genes from HIV-1(SF162) (R5, MT/NSI) in the context of the molecular clone SIVmac239. This virus induces immunosuppression, leading to the development of AIDS in macaques. Twelve macaques were screened and found to be seronegative for simian T-lymphtropic virus, SHIV, SIV, B virus, and Type D retroviruses. Eight SHIV-infected macaques (*#E1-E8*) were inoculated intravenously at 40 weeks of age with SHIV-_SF162.P4_ (provided by Dr. Nancy Miller, at the National Institute of Allergy and Infectious Diseases, National Institutes of Health, Bethesda, MD, USA), and were sacrificed at 28 weeks after inoculation (Table 1). Four uninfected macaques (*#10-13*) were used as controls (Table 1). The animals were housed in individual cages and maintained according to the rules and guidelines of the Institute of Laboratory Animal Sciences of Chinese Academy of Medical Science, and the National Institute for Infectious Diseases and Experimental Animal Welfare. The protocol was approved by the Committee on the Ethics of Animal Experiments of the Institute of Laboratory Animal Sciences of Chinese Academy of Medical Science, China. The animals were sacrificed at same time after infection, when they became morbid, as previously described [6, 33–35].

### Viral RNA loads

Viral RNA loads in the peripheral blood of the eight SHIV-infected macaques were measured at the time of autopsy (Table 1).

### Histopathological examination

Test brain tissues were derived from the frontal cerebral cortex of 12 macaques, including eight from the SHIV-infected group and four from the normal control group. Paraffin-embedded brain tissue sections (5 μm thick) were used. Immunohistochemistry was performed using a streptavidin peroxidase (SP) kit (Maixin Biotechnology, China, KIT9720) with antibodies specific to excitatory amino acid transporter-2 (EAAT-2) (1:5000; Millipore, Germany, AB1783), neuron-specific nuclear protein (NeuN) (1:200; Millipore, ABN78), cleaved-caspase-3 (1:1000; Cell Signaling, USA, 9661), astrocyte-elevated gene 1 (AEG-1) (1:200; Abcam, USA, ab131291), glial fibrillary acidic protein (GFAP) (1:200; Millipore AB5541), ionized calcium-binding adaptor molecule 1 (Iba1) (1:200; Wako Chemicals, Japan, 019-19741), respectively, strictly according to manual instructions.

### Double-labeling immunohistochemistry

To determine neuronal apoptosis in the cerebral cortex of SHIV-infected rhesus, we performed double-labeling immunohistochemistry using the Envision method, first for NeuN (1:200) using DAB/peroxidase (PO) (Maixin Biotechnology, China, DAB0031), and then for cleaved-caspase-3 (1:1000) using PermaBlue Plus/alkaline phosphatase (AP) (Diagnostic Biosystems, Japan, K058). To determine the relationship between decreased EAAT-2 expression and apoptosis, we performed double-labeling immunohistochemistry for EAAT-2 (1:5000) using DAB/PO, and for cleaved-caspase-3 (1:1000) using PermaBlue Plus/AP.

To determine the phenotype of AEG-1-positive cells, we performed double-labeling immunohistochemistry, first for AEG-1 (1:200) using AEC/PO (Maixin Biotechnology, China, AEC0037) or PermaBlue Plus/AP, and then for GFAP (1:200), NeuN (1:200) and Iba-1 (1:200), using PermaBlue Plus/AP or DAB/PO.

To determine the relationship between decreased EAAT-2 expression and increased AEG-1 expression, we performed double-labeling immunohistochemistry, first for EAAT-2 (1:5000) using DAB/PO, and then for AEG-1 (1:200) using PermaBlue Plus/AP.

### Cell cultures

Astrocytes in primary culture were prepared from 1- to 2-day-old C57BL wild type mice and were cultured in DMEM/F12+GlutaMAX^tm^ (Gibco, Life Technologies, Madrid, Spain, 10565018) supplemented with 10% FBS (Gibco, 12657-029). The U87 cell line was purchased from American Type Culture Collection (Manassas, VA, USA) and was cultured in DMEM (Gibco, C11995500BT) supplemented with 10% FBS.

### Cell transfection

The pRK5M-Tat-flag or pcDNA3.1-AEG-1-myc overexpression plasmids, as well as the empty vector, were prepared in the *E. coli* DH5α strain and extracted using an Endofree Maxi plasmid kit (Tiangen, China, DP117).

For the AEG-1 knockdown experiment, we designed specific and scrambled sequences for AEG-1 cDNA from different areas (Table 2). Plasmid-2 was used for subsequent experiments since western blot showed it inhibited AEG-1 more strongly than the other plasmids.

**Table 2:** Primers for AEG-1 interference

Name	Forward primer (5′-3′)	Reverse primer (5′-3′)
Pll3.7-AEG-1-shRNA	TGACCATGAGCACTAGTGACTTCAAGA GAGTCACTAGTGCTCATGGTCTTTTTTC	TCGAGAAAAAAGACCATGAGCACTAGTGA CTTCAAGAGAGTCACTAGTGCTCATGGTCA
	TGCGTGATGATAACGTAGGTCTATTCAAGA GATAGACCTACGTTATCATCACGTTTTTTC	TCGAGAAAAAAGCGTGATGATAACGTAGG TCTATTCAAGAGATAGACCTACGTTATCATCACGA
	TGACACTGGTGACACTAATGTTCAAGA GACATTAGTGTCACCAGTGTCTTTTTTC	TCGAGAAAAAAGACACTGGTGACACTAATG TTCAAGAGACATTAGTGTCACCAGTGTCA

U87 cells were seeded into 6-well plates and cultured with complete DMEM supplemented with 10% fetal bovine serum (FBS) at 37°C and 5% CO_2_ in a humidified incubator. After being allowed to adhere, U87 cells were transfected with pRK5M-Tat-flag (3 μg), pcDNA3.1-AEG-1-myc (3 μg), empty vector (3 μg), Pll3.7-AEG-1-shRNA (3 μg), or Pll3.7-scamble-shRNA (3 μg) using Turbofect Reagent (3 μl) (Thermo Scientific, USA, R0532) according to manufacturer protocols. After 4-6-h incubation, the cultured medium was completely replaced with fresh DMEM and 10% FBS, and cells were further incubated for another 48 h in 6-well plates after which they were extracted for western blot analysis.

### Western blot analysis

Primary cultures of mice astrocytes or glioma U87 cells were cultured in 6-well plates and treated for 48 h with varying stimuli prior to extraction. Total protein was extracted using RIPA lysis buffer (Boster, China, AR0105-100) and then quantified using the BCA Protein Assay Kit (Thermo Scientific Pierce, Rockford, IL, USA, 23228). From each sample, 15 μg of protein were loaded and separated by a 10-15% sodium dodecyl sulfate polyacrylamide gel electrophoresis gradient, and subsequently transferred to a nitrocellulose membrane. Membrane-bound proteins were blocked with 5% milk powder for 1 h, and washed with phosphate-buffered saline Tween-20 (PBST) four times at room temperature after blocking. The membranes were then incubated with primary antibodies against various proteins, including HIV-1 Tat (1:1000; Abcam, USA, ab63957), AEG-1 (1:1000; Abcam, ab131291), YY-1 (1:1000; Cell Signaling, 2185), EAAT-2 (1:1000; Cell Signaling, 3838), P65 (1:1000; Product Description, USA, 10745-1-AP), or GAPDH (1:10000; Abcam, ab181602) overnight at 4°C, followed by washing four times with PBST, incubating with horseradish peroxidase-labeled secondary antibodies (1:1000; R&D Systems, USA, HAF008) for another 1 h at room temperature, and washing four times with PBST. Chemiluminescent HRP substrate (Millipore, Germany, WBKLS0500) was used to visualize the protein bands. The molecular weights of HIV-1 Tat, AEG-1, YY-1, EAAT-2, P65, and GAPDH were 20 kDa, 65 kDa, 65 kDa, 65 kDa, 65 kDa, and 37 kDa, respectively.

### Immunofluorescence

A round slide was placed into a 24-well plate and polylysine was used for coating. The U87 cells were inoculated onto the round slide within the 24-well plate, and HIV-1 Tat (100 ng/ml; Prospec, USA, hiv-105) was added and incubated for 48 h after cell attachment. After treatment, U87 cells were fixed in 5% paraformaldehyde for 20 min at room temperature and then blocked with blocking buffer (5% BSA in 1X PBS containing 0.1% Triton X-100) for 1 h. The samples were then incubated with primary antibodies specific to AEG-1 (1:100) in blocking buffer overnight at 4°C, washed four times with PBS, and incubated with fluorescent secondary antibodies (goat anti-rabbit-647 nm; Abcam, ab150079) for 1.5 h. The round slide was carefully removed and stained with DAPI for nuclear staining. Finally, images were collected under a fluorescence microscope (Nikon, Japan, Eclipse Ti).

### Quantitative analysis of AEG-1 and EAAT-2 mRNA expression by real-time PCR

Total RNA was extracted from U87 cells using Trizol Reagent (Life Technologies, 15596018). Total RNA (1 μg) from each sample was used to synthesize cDNA using the ReverTra Ace qPCR RT Master Mix (Toyobo, Japan, FSQ-201), then amplified using the FastStart Universal SYBR Green Master (ROX) (Toyobo, 11750800). qRT-PCR was performed in triplicate tubes on the AB 7100 RT-PCR system (Applied Biosystems, USA). qRT-PCR was performed as follows: one cycle at 95°C for 15 min and 40 amplification cycles (94°C for 15 s, 1 min at 55°C, and 70°C for 30 s). Sample fluorescence was measured during the annealing step. Fluorescence data were continuously collected to obtain the dissociation curve. Fluorescence was plotted *versus* Ct (threshold cycle) based on dRn (baseline-corrected, reference dye-normalized fluorescence) to obtain the standard curve and to measure initial template quantity. The average Δ-Δ cycle threshold of different samples was analyzed to calculate the relative mRNA levels, using β-actin mRNA as the control. Fluorescence data were analyzed using GraphPad Prism 5.0 Software (GraphPad Software, USA). The forward and reverse primers used for each gene are shown in Table 3.

**Table 3:** Primers for the RT-PCR Assay

Name	Forward primer (5′-3′)	Reverse primer (5′-3′)
AEG-1	AAATGGGCGGACTGTTGAAGT	CTGTTTTGCACTGCTTTAGCAT
EAAT-2	CCTGACGGTGTTTGGTGTCAT	CAAGCGGCCACTAGCCTTAG
β-actin	CATGTACGTTGCTATCCAGGC	CTCCTTAATGTCACGCACGAT

### Statistical analysis

Data were expressed as mean ± standard deviation (SD) from at least three separate experiments. Significance of differences between groups was determined using the unpaired *t*-test. Results were considered statistically significant if *P* < 0.05 according to analysis of variance.
